# Neuronal cell death mechanisms in Alzheimer’s disease: An insight

**DOI:** 10.3389/fnmol.2022.937133

**Published:** 2022-08-25

**Authors:** Parul Goel, Sasanka Chakrabarti, Kapil Goel, Karanpreet Bhutani, Tanya Chopra, Sharadendu Bali

**Affiliations:** ^1^Department of Biochemistry, Shri Atal Bihari Vajpayee Government Medical College Chhainsa, Faridabad, India; ^2^Department of Biochemistry, Maharishi Markandeshwar Institute of Medical Sciences and Research, Maharishi Markandeshwar (Deemed to be University), Ambala, India; ^3^Department of Community Medicine and School of Public Health, Post Graduate Institute of Medical Education and Research, Chandigarh, India; ^4^Department of Surgery, Maharishi Markandeshwar Institute of Medical Sciences and Research, Maharishi Markandeshwar (Deemed to be University), Ambala, India

**Keywords:** Alzheimer’s disease, apoptosis, necroptosis, autophagy, ferroptosis

## Abstract

Regulated cell death (RCD) is an ordered and tightly orchestrated set of changes/signaling events in both gene expression and protein activity and is responsible for normal development as well as maintenance of tissue homeostasis. Aberrant activation of this pathway results in cell death by various mechanisms including apoptosis, necroptosis, pyroptosis, ferroptosis, and autophagy-dependent cell death. Such pathological changes in neurons alone or in combination have been observed in the pathogenesis of various neurodegenerative diseases including Alzheimer’s disease (AD). Pathological hallmarks of AD focus primarily on the accumulation of two main protein markers: amyloid β peptides and abnormally phosphorylated tau proteins. These protein aggregates result in the formation of A-β plaques and neuro-fibrillary tangles (NFTs) and induce neuroinflammation and neurodegeneration over years to decades leading to a multitude of cognitive and behavioral deficits. Autopsy findings of AD reveal massive neuronal death manifested in the form of cortical volume shrinkage, reduction in sizes of gyri to up to 50% and an increase in the sizes of sulci. Multiple forms of cell death have been recorded in neurons from different studies conducted so far. However, understanding the mechanism/s of neuronal cell death in AD patients remains a mystery as the trigger that results in aberrant activation of RCD is unknown and because of the limited availability of dying neurons. This review attempts to elucidate the process of Regulated cell death, how it gets unregulated in response to different intra and extracellular stressors, various forms of unregulated cell death, their interplay and their role in pathogenesis of Alzheimer’s Disease in both human and experimental models of AD. Further we plan to explore the correlation of both amyloid-beta and Tau with neuronal loss as seen in AD.

## Introduction

Alzheimer’s disease is a chronic neurodegenerative disease of the elderly people recognized clinically as gradual impairment in memory and cognition along with various disorders in the emotional domain (Sperling et al., 2011; Scheltens et al., 2016; Qiu et al., 2022). It is considered the sixth leading cause of death in the United States where the death from AD increased by 71% from 2000 to 2013 (Alzheimer’s Association, 2016). Estimated in 2015, the burden of dementia worldwide constitutes over 46 million affected people and a societal loss of United States dollar 818 billion, and AD accounts for the vast majority of such patients with dementia (Prince et al., 2015). The disease usually presents in a sexagenarian and follows a progressive and irreversible course. As the memory and cognitive skills continue to get worsened, the patients eventually depend on caregivers extensively and finally succumb to the illness within 4–8 years after diagnosis. Changes in the brain related to AD, however, begin in the preclinical AD stage several decades before the manifestation of clinical dementia (Dubois et al., 2016). The vast majority of AD subjects suffer from the sporadic form of the disease, while in less than 5% of cases the disease appears in the familial form with defined mutations in *APP, PS1*, and *PS2* genes (Reitz and Mayeux, 2014). Neuropathologically, the AD brain is characterized by extra-cellular depositions of oligomerized and fibrilar amyloid beta peptide 42 (Aβ42) surrounded by dystrophic neurites called neuritic plaques, as well as intra-neuronal formation of neurofibrillary tangles composed of phosphorylated tau protein (Serrano-Pozo et al., 2011; Chakrabarti et al., 2015). Associated with these changes diffuse neuronal loss, synaptic degeneration and reactive gliosis could be seen in many brain areas concerned with memory and cognition (Serrano-Pozo et al., 2011; Chakrabarti et al., 2015).

Aging is considered as the primary risk factor for many neurodegenerative diseases including AD, and after 65 years the age-specific prevalence of AD almost doubles every 5 years (Harman, 2006; Qiu et al., 2022). The factors responsible for making certain individuals progress from non-pathological brain aging to complex cascade of AD pathogenesis are largely unknown, but the commonality of underlying mechanisms in these two processes are interesting (Podtelezhnikov et al., 2011; Poddar et al., 2019). Apart from aging, multiple genetic and modifiable risk factors also contribute to the pathogenesis of sporadic AD (Reitz and Mayeux, 2014; Qiu et al., 2022). For more than two decades, the dominant hypothesis attempted to explain the pathogenesis of AD by the multiple toxic effects of oligomerized Aβ42 on neurons, glia and different subcellular components in the brain (amyloid cascade hypothesis), but the shortcomings of this hypothesis are becoming increasingly apparent (Korczyn, 2008; Herrup, 2015). It appears that at the cellular level in the AD brain, mitochondrial dysfunction, oxidative stress, abnormal accumulation of proteins (Aβ42, tau, etc.) with associated toxic effects and inflammatory response by microglia interact in a concerted manner mediating the pathogenesis of AD (Chakrabarti et al., 2015; Ganguly et al., 2017; Guo et al., 2020). This review will, however, not cover the different aspects of AD pathogenesis, but focus on a limited aspect of the latter scrutinizing the available evidence on the nature and mechanisms of neuronal death in AD, highlighting the knowledge gaps and providing new insights into this complex process.

## Neuronal death in Alzheimer’s disease brain

The high degree of neuronal death apparent in various areas of the brain in AD contrasts with relatively little neuronal loss in non-pathological brain aging (West of Scotland Coronary Prevention Group, 1996). The neuronal loss in AD begins in the preclinical stage and then progresses during the prodromal phase or MCI and AD dementia; an early loss could be in the entorhinal cortex, basal nucleus of Meynert, locus ceruleus, but later the neuronal loss is diffusely present in multiple brain areas like the hippocampus, amygdala, temporal lobe and frontal and parietal cortices (Gómez-Isla et al., 1996, 1997; Serrano-Pozo et al., 2011; Arendt et al., 2015; DeTure and Dickson, 2019). It is not certain if the neuronal loss begins in one brain area and then progresses to other areas or starts simultaneously in different regions. The neuronal loss correlates well with the regional distribution of NFTs, but within a defined region the neuronal loss is more conspicuous than the presence of NFTs (Serrano-Pozo et al., 2011). Interestingly, synaptic degeneration might precede neuronal loss, and both these processes contribute to memory impairment in AD patients (Arendt et al., 2015). Although multiple studies have shown that the cognitive deficits correlate with the extent of NFT formation, it is even better correlated with the degree of neuronal loss and synaptic degeneration (Serrano-Pozo et al., 2011; Arendt et al., 2015). In multiple studies the neuronal loss has been estimated in transgenic AD models (carrying mutated familial AD genes or Tau gene) in brain areas affected in clinical AD especially the hippocampus; in single APP transgenic or double or triple transgenic (*APP/PS1* or *APP/PS2* or *APP/PS1/TAU*, etc.) experimental animals a slight to substantial neuronal loss was observed (Schmitz et al., 2004; Wirths and Bayer, 2010; Wirths and Zampar, 2020). However, the neuronal death is much less pronounced than amyloid-beta pathology or tauopathy or reactive gliosis in the brains of AD transgenic animals (Wirths and Zampar, 2020). Before analyzing further this aspect of neuronal death in AD, it would be worthwhile to provide a bird’s eye view of different pathways of cell death and salient features of neuronal differentiation and death in the brain.

### Cell death types: A snapshot

Cell death can be activated in response to various forms of internal or cellular stress, and primarily it is classified into two major groups- accidental cell death and regulated cell death (RCD). The physiological form of regulated cell death is programmed cell death (Cui et al., 2021). RCD has been sub-classified over the years from the early characterization of apoptosis in more than ten sub-types of ingenious names (Tang et al., 2019). However, we will limit ourselves to a discussion on some of these pathways that appear to be more relevant in the context of AD.

#### Apoptosis

Apoptosis is a type of regulated cell death (RCD) characterized by morphological features that include cell shrinkage, chromatin condensation, nuclear fragmentation, plasma membrane blebbing and the formation of membrane-bound apoptotic bodies (Elmore, 2007; Toné et al., 2007; Del Re et al., 2019). The pathway of apoptosis can be triggered by the binding of extracellular death ligands (TNF-α, FASL, TRAIL, etc.) to death receptors (extrinsic pathway); on the other hand, mitochondrial membrane depolarization as a result of cellular injury (DNA damage, metabolic stress, ROS) might initiate the intrinsic pathway of apoptotic death (Elmore, 2007; Galluzzi et al., 2012; Tang et al., 2019). In both the pathways a group of proteolytic enzymes known as caspases, derived from the inactive forms called pro-caspases, are involved, and some of these are initiator caspases (caspase 8, caspase 9, caspase 10) and others effector caspases (caspase 3, caspase 6, caspase 7) (Elmore, 2007; Del Re et al., 2019). The effector caspases degrade various protein substrates to cause the cytomorphological and functional alterations of apoptosis; the chromatin fragmentation is because of activation of CAD following the degradation of ICAD which keeps the CAD as an inactive complex (Elmore, 2007; Cui et al., 2021). However, a caspase-independent intrinsic apoptotic pathway is also known (Galluzzi et al., 2012). The mitochondria play a central role in the intrinsic pathway of apoptosis. The mitochondrial outer membrane permeabilization (MOMP) mediated by pro-apoptotic proteins BAX and BAD of the Bcl-2 family is a key event of intrinsic apoptosis, but mitochondrial inner membrane permeability alterations and loss of transmembrane potential are also involved in this process (Elmore, 2007; Galluzzi and Kroemer, 2008; Wang and Youle, 2009; Averill-Bates et al., 2018; Tang et al., 2019; Cui et al., 2021). These membrane changes lead to the release of cytochrome c, AIF, endoG, Smac/DIABLO, etc., from the mitochondria causing activation of effector caspases and apoptotic death (Averill-Bates et al., 2018; Del Re et al., 2019; Tang et al., 2019).

In the extrinsic pathway, the activation of initiator caspase 8 occurs which might further activate executioner caspase like caspase 3 leading to apoptotic death; alternatively, a cross-talk between extrinsic and intrinsic pathways takes place in which caspase 8 acts on BID forming the truncated BID (tBID) which might lead to mitochondrial MOMP with subsequent initiation of intrinsic apoptotic cascade (Elmore, 2007; Tang et al., 2019).

#### Necrosis and necroptosis

Traditionally, necrosis is considered an unregulated cell death process characterized by cell swelling, loss of membrane integrity, dissipation of ionic gradients, and release of intracellular contents into the extracellular environment; the necrosis usually triggers an inflammatory response presumably through the release of DAMPs or ‘damage-associated molecular patterns’ from the dead cells (Vanlangenakker et al., 2012; Feoktistova and Leverkus, 2015; Sachet et al., 2017). Necrosis can occur due to overwhelming external physicochemical stress such as freezing, burning, mechanical trauma, and this is often categorized as accidental or primary necrosis; a process of secondary necrosis has also been suggested as the outcome of apoptosis under certain conditions where the clearance of apoptotic cells by phagocytes is absent or impaired (Silva, 2010; Vanlangenakker et al., 2012; Feoktistova and Leverkus, 2015; Sachet et al., 2017). The necrosis generally lacks a definite cellular signaling pathway, but some forms of necrosis follow well-defined mechanisms such as the one driven by mitochondrial permeability transition pore (mPTP) activation (Kinnally et al., 2011; Feoktistova and Leverkus, 2015). On the other hand, necroptosis refers to a regulated form of cell death with morphological features of necrosis but with a defined signaling cascade involving RIP kinases (RIPK1 and RIPK3), necrosome formation and activation of MLKL (Berghe et al., 2010; Galluzzi et al., 2018). Necroptosis can be induced by the ligand-binding to several death receptors (TNFR1, TRAIL-R1 and -R2, FAS), interferon receptors and other receptors like stocktickerRIG1, STING sensing pathogen-associated molecular patterns; in addition activated TLRs or interferon α-receptor and certain viruses can trigger necroptosis without involving RIPK1 (Tummers and Green, 2017; Choi et al., 2019; Tang et al., 2019). In the typical necroptotic pathway such as the one induced by TNF-α, the ligand binding initiates a multimeric complex formation with RIPK1 (complex I) followed by the formation of another complex called complex II within which autophosphorylation of RIPK1 occurs at serine 161; the activated RIPK1 then phosphorylates RIPK3 and forms a complex with it called necrosome (Vanlangenakker et al., 2012; Su et al., 2016; Choi et al., 2019). A subsequent phosphorylation of MLKL by RIPK3 causes the oligomerization of MLKL that culminates in lytic cell death (Vanlangenakker et al., 2012; Su Z. et al., 2016; Choi et al., 2019). It is important to realize that the same stimuli that activate the extrinsic pathway of apoptosis might also trigger necroptosis, and this presumably occurs when caspase 8 is deficient or its activation is impaired (Choi et al., 2019; Tang et al., 2019). Likewise, the deficiency of FADD or cIAPs might also direct the cell to follow necroptosis instead of apoptosis (Vanlangenakker et al., 2012; Choi et al., 2019). Necroptosis can be inhibited by necrostatin which is a specific inhibitor of RIPK1.

#### Ferroptosis

The ferroptotic mode of cell death is iron-dependent and induced by diverse triggers; it involves an accumulation of ROS and lipid peroxidation products and a depletion of reduced glutathione inside the cells followed by cell death, and the phenomenon is prevented by iron-chelators, vitamin E, butylated hydroxytoluene, and several other inhibitors of lipid peroxidation like ferrostatin-1 and liproxstatin-1 (Stockwell et al., 2017; Li J. et al., 2020). Morphologically, ferroptosis is associated with shrunken mitochondria with loss of cristae and rupture of the outer mitochondrial membrane, chromatin condensation, cytoplasmic swelling and cell membrane rupture (Li J. et al., 2020). The term ferroptosis was initially identified as a mode of non-apoptotic death triggered by erastin in fibrosarcoma cells; erastin inhibits the transport of cystine through cystine/glutamate antiport system leading to a deficient intracellular antioxidant status as a result of decreased content of reduced glutathione (Dixon et al., 2012). Many other molecules have been shown to induce ferroptosis which includes glutathione peroxidase 4 (Gpx4) inhibitor like RSL3, BSO or buthionine sulfoximine causing cellular glutathione depletion, compounds increasing intracellular iron content and inhibitors of mitochondrial electron transport chain (Cao and Dixon, 2016; Xie et al., 2016; Xu et al., 2019). Genetic studies have identified further that apart from Gpx and cystine/glutamate antiport, cellular iron transporters, iron-responsive element binding proteins, ferritin degradation, acyl-CoA synthetase long-chain family member 4 (ACSL4), lysophosphatidyl acyltransferase 3, NRF-2 pathway, etc., are important modulators of ferroptosis which have been implicated in various pathological conditions including cancer and neurodegenerative diseases (Cao and Dixon, 2016; Xu et al., 2019; Li J. et al., 2020). Despite initial ambiguities, disruption of the plasma membrane and ROS production from NADPH oxidase, mitochondrial respiratory complexes and iron-catalyzed Fenton’s reaction are all considered important features of ferroptosis (Dixon et al., 2012; Xie et al., 2016; Xu et al., 2019).

#### Autophagy

Autophagy or macroautophagy is primarily a mechanism by which damaged and dysfunctional organelles or subcellular components, protein aggregates are degraded by the cell by forming a double-membrane structure (autophagosome) which fuses with the lysosomes for the final degradation of its content by the lysosomal hydrolytic enzymes (Green and Llambi, 2015; Araújo Pereira et al., 2016; Cui et al., 2021). There is some debate about whether autophagy is a pro-survival event or a cell death pathway; probably the autophagy in certain contexts functions as a cell death pathway with typical morphological features of cytoplasmic vacuolization and lysosomal degradation (Green and Llambi, 2015). Though initially characterized in yeast, many autophagy related (Atg) proteins have been identified in mammalian system which through complex interactions help in the formation and maturation of autophagosome and its subsequent fusion with lysosomes (Green and Llambi, 2015; Araújo Pereira et al., 2016; Galluzzi et al., 2017). Thus Beclin1 (Atg6), Vsp34, Vsp15 form an initiation complex which with the help of several Atg proteins finally causes conjugation of LC3 with phosphatidyl ethanolamine forming LC3II which assists in the fusion of autophagosomes with lysosomes resulting in the formation of autolysosomes; the latter process also requires other proteins especially the transmembrane LAMP2B present in the lysosomal membrane (Badadani, 2012; Green and Llambi, 2015; Galluzzi et al., 2017). Various cellular stressors such as nutrient deprivation, hypoxia, ER-stress and absence of growth factors can regulate autophagy by modulating the activities of PKA, AMPK or mTORC1 (Parzych and Klionsky, 2014; Galluzzi et al., 2017). A special type of autophagy called microautophagy simply involves the direct engulfment of autophagic substrates by invagination of the lysosomal membrane and the subsequent degradation of the captured cargo by the hydrolytic enzymes (Parzych and Klionsky, 2014; Galluzzi et al., 2017). Chaperone-mediated autophagy or CMA is distinct from the macroautophagy and involves the removal of specific proteins containing a KFERQ-like pentapeptide motif which can bind the molecular chaperone such as the heat shock cognate 70 kDa protein or hsc70; several co-chaperones like hsp90, hsp40, Bag-1 co-operate with this process (Bejarano and Cuervo, 2010; Galluzzi et al., 2017). The hsc70 facilitates the translocation of the CMA substrate proteins through lysosomal membranes with the help of the LAMP2A receptor (Bejarano and Cuervo, 2010; Galluzzi et al., 2017).

#### Pyroptosis

This is a form of lytic cell death, morphologically distinct from apoptosis and necrosis, which is associated with inflammation; the formation of inflammasomes, the activation of inflammatory caspases such as caspase-1/4/5/11, cleavage of the Gasdermin family of proteins especially Gasdermin-D, maturation and release of various inflammatory cytokines IL-1β and IL-18 at different phases of pyroptosis (Tan et al., 2014; Fang et al., 2020; Cui et al., 2021; Pian et al., 2021). Pyroptosis can take place either independent of inflammasome formation or by an inflammasome dependent mechanisms. When the process is independent of inflammasome formation, there is an early activation of caspase-3 which cleaves Gasdermin-E, and the N-terminal portion of Gasdermin-E oligomerizes to form a pore through the cell membrane initiating the pyroptotic death of the cells and further, the N-terminal portion of Gasdermin-E can activate the inflammasome-mediated pyroptosis (Tan et al., 2021). In other contexts, the granzyme proteases might be activated to cleave Gasdermin-B and its N-terminal portion may again form membrane pores (Tan et al., 2021). On the other hand, pyroptosis occurs through the mediation of inflammasomes either following a canonical pathway or a non-canonical pathway. In the canonical pathway, several members of NOD-like receptors (NLRP1, NLRP3, NLRC), pro-caspase-1 and other proteins such as apoptosis-associated spec-like protein containing a CARD (ASC) form a multimeric complex of inflammasome where pro-caspase-1 is cleaved to active caspase-1; the active caspase-1 helps in the maturation of IL-1β and IL-18 by proteolytic cleavage and also releases the N-terminal portion of Gasdermin-D which oligomerizes to form membrane-spanning pores (Hsu et al., 2021; Pian et al., 2021). In the non-canonical pathway inflammatory caspases (caspase-4/5/11) are activated by intracellular lipopolysaccharide or LPS, and the active caspases then cleave Gasdermin-D triggering the formation of membrane-pores (Pian et al., 2021; Tan et al., 2021). The inflammasome mediated pyroptosis is initiated by the cytosolic receptors that recognize pathogen-associated molecular patterns or danger-associated molecular patterns in the form of bacterial or viral or fungal peptides, lipopolysaccharides, β-glucan, nucleic acids or toxins or sometimes endogenous molecules (Hsu et al., 2021; Pian et al., 2021; Tan et al., 2021). A body of experimental evidence has shown that pyroptosis is important in the context of innate immunity, inflammatory response and tumor suppression and cancer therapy; additionally, it might be a key mechanism of many diseases like cerebral ischemia, myocardial infarction, neurodegenerative diseases and others where inflammation is an important component of pathology (Tan et al., 2014; Fang et al., 2020; Cui et al., 2021).

### Neuronal death during development and disease

Regulated cell death signaling cascades in the brain are active during the embryonic developmental phase as well as in the early post-natal period; these pathways operate in both mitotic precursors of neurons and post-mitotic differentiated neurons in a spatially and temporally restricted manner in removing the surplus neurons, providing shape to the CNS and establishing the normal neural architecture and circuitry (Kim and Sun, 2011). The major RCD mechanism responsible for normal neural development in both embryonic and post-natal phase is apoptosis (Yamaguchi and Miura, 2015). However, in the adult brain; the cell death pathways become highly restricted, because the mature neurons utilize multiple complex mechanisms to inhibit the activation of cell death pathways (Kole et al., 2013; Hollville et al., 2019). However, the triggers and pathological changes that cause reversal of events that normally promote the survival of mature neurons are yet to be fully established. Kole et al. (2013) further stated that return to immature neuronal phenotype might be a fundamental mechanism in various neurodegenerative disorders known for progressive and widespread neuronal death. In a recent study by Mertens et al. (2021), an age-equivalent neuronal model was generated by direct conversion of fibroblasts of AD patients into induced neurons and transcriptome from such induced neurons (AD iNs) displayed immature and upregulated expression of progenitor- like signaling pathways and downregulation of properties of mature neurons. Further, these iNs reflected a hypo-mature state as they exhibited various markers of stress, de-differentiation and cell cycle re-entry, and based on these observations the authors concluded that these changes are responsible for the many pathological changes seen in neurons in AD brains. Using neurons from EOFAD patients; Caldwell et al. (2020) generated human induced pluripotent stem cell (hiPSC) and demonstrated their dedifferentiation to precursor-like state with markers of ectoderm and non-ectoderm lineages.

García-Osta et al. (2022) in their recent review highlighted that in response to various external stimuli (Aβ42, p-tau, insulin resistance) the neurons try to re-enter mitosis but there is abortive re-entry into the cell cycle that ultimately results in neuronal degeneration. Re-expression of cyclins in post mitotic neurons represents reactivation of the cell cycle and re-entry of terminally differentiated neurons; which is implicated in the pathogenesis of AD (Sharma et al., 2017). Evidence from post mortem AD brains and (iPSC)-derived neurons indicate that in the early stages of AD; neurons show various signs of de-differentiation and return to progenitor-like fates, re-enter cell cycle, become dysfunctional and ultimately lose their resilience (Koffie et al., 2011; Arendt, 2012; Kole et al., 2013). Further recent evidence from -omics data on AD patients reveals abnormal activation of various cell cycle components to be responsible for neuronal death in AD (Hooper et al., 2020). Increased levels of Cyclin E in the hippocampal region of AD brains than in age-matched controls suggest that in AD, the cell cycle is driven through the G1/S phase and this was found to match with plaque burden in the neocortex region (Bonda et al., 2010). Furthermore, in various studies on animal models, improvement in symptoms of AD was noted after administration of CDKIs (Senderowicz, 1999; Leggio et al., 2016; Wilkaniec et al., 2018; Zhang et al., 2019; Allnutt et al., 2020; Malhotra et al., 2021). To obviate the disease symptoms in such disorders; various biomolecules – Flavopiridol, Roscovitine, and Olomoucine as CDKs inhibitor, flavonoids – Resveratrol, Apigenin and Epigallocatechin-gallate for inducing cell cycle arrest at different checkpoints is used as drug for the treatment of AD and other neurodegenerative diseases (Sharma et al., 2017; Malhotra et al., 2021). Modi et al. (2015) in their study on transgenic mouse model of AD used miR-34a to regulate neuronal cell cycle by suppressing cyclin D1 thus highlighting the importance of cell cycle suppression for survival of terminally differentiated neurons.

Traxler et al. (2021) in their recent review highlighted that under pathogenic metabolic states there is a loss of resilience and re-activation of cell death mechanisms in neurons unlike the somatic cells which shift to malignant transformation. Neurons rely heavily on mitochondrial oxidative phosphorylation for energy as they require a large amount of energy to maintain ion gradients and generate action potentials with negligible proportion coming from glycolysis (Mergenthaler et al., 2013; Zhang et al., 2014; Zheng et al., 2016). However, based on several lines of evidence Traxler et al. (2021) in their review concluded that the metabolic switch from oxidative phosphorylation to glycolysis as a primary source of energy in neurons renders them susceptible to cell death as they reverse to precursor-like state. A simple activation of glycolysis in mature neurons has been suggested to act as a trigger for neuronal cell death (Herrero-Mendez et al., 2009). This metabolic switch leads to neuronal de-differentiation whereby the mature neurons return to immaturity and reach such a point at which they become apoptosis competent again (Kole et al., 2013; Su et al., 2016). Post-mortem brain tissue from AD patients further showed signs of metabolic state switch from oxidative phosphorylation to glycolysis in neurons and thus support the fact of neuronal de-dedifferentiation (Marinaro et al., 2020).

Though multiple evidence from different studies shows increased expression of immaturity markers in mature neurons derived from brains of patients with AD and other neurodegenerative diseases (Nagy et al., 1997; Jin et al., 2004; Koffie et al., 2011; Kole et al., 2013); this is not the case as contrary findings have been observed in different sets of experiments (Hagihara et al., 2019). Tobin et al. (2019) observed no difference in immaturity markers in AD vs. control brains while on the other hand decreased expression of immaturity markers in AD brains was noted by another group (Moreno-Jiménez et al., 2019). Both these observations raise strong doubts on the hypothesis of increase/upregulation in dematuration/cell cycle re-entry in AD patients. Based on these completely different set of observations, Hagihara et al. (2019) in a review concluded that re-expression of immaturity/cell cycle markers on mature neurons must be interpreted with caution.

### Types of neuronal death in Alzheimer’s disease

#### Post-mortem studies

##### Apoptosis

The previous report by Lassmann et al. (1995) illustrates 30 times higher DNA fragmentation in brain tissue from AD cases than from age-matched controls. Smale et al. (1995) observed increased apoptosis in the hippocampal area in brains of AD patients as compared to age-matched non-AD brains. Further apoptosis was observed in both neurons and astrocytes. Levels of various apoptotic, pro-apoptotic and anti-apoptotic proteins were examined in cytosolic and membranous fractions of temporal cortex from AD patients and control brains and increased levels of Bcl-2 alpha, Bcl-xL, Bcl-x beta, Bak and Bad in membranous fraction, Bcl-x beta in cytosolic fraction were observed (Kitamura et al., 1998). In another study, Troncoso et al. (1996) observed TUNEL positivity in neurons, glia and microglial cells in AD brains than age-matched controls and highlighted the laminar pattern of this process in the neocortical region. On the other hand; Selznick et al. (1999) in their study noted no association of senile plaques and NFTs in cortical and hippocampal brain sections from AD patients with caspase-3 activation and concluded non-significant role of apoptosis in neuronal death seen in AD. In comparison of neurons from AD and age-matched controls, Stadelmann et al. (1999) observed caspase-3 immunoreactivity in AD neurons and in GVD granules and concluded that in AD, neuronal apoptosis progresses at a slow rate. Contrary to these findings; Su et al. (2001) studied elevated caspase 3 immuno neurons and astrocytes from AD brains and also noted its co-localization with senile plaques and NFT. Matsui et al. (2006) observed elevated mRNA levels of caspase 7 and 8 in the temporal neocortex of AD brains in comparison to control while no significant difference was detected in mRNA levels of caspase 3 and 9. The authors also noted the correlation of Aβ42 accumulation with transcriptional activation of apoptotic cascade components (Matsui et al., 2006). Based on the observation of immunoreactivity of Caspase 6 in neuritic plaques and NFTs, Albrecht et al. (2009) highlighted the role of caspase-6 in pathophysiology of AD. Burguillos et al. (2011) observed activation of caspase-8 and caspase-3/7 in microglia from the frontal cortex of AD brains and concluded that caspase signaling controls both microglia activation and neurotoxicity. Neuroinflammation mediated by microglia plays a significant role in neurotoxicity. Immunohistochemical analysis of hippocampal tissue sections from AD cases and controls by Rohn et al. (2001) revealed immunolabeling with CASP-8p18 in neurons of AD patients suggesting the role of caspase-8 in activation of caspase 3 inside the neurons of AD patients. Anderson et al. (1996) performed double labeling experiments on AD tissue to explore the association of c-Jun with DNA strand breaks and noted strong co-localization between these two markers, however gel electrophoresis of DNA isolated from AD and control brain tissue did not reveal any evidence of apoptotic or necrotic cell death mechanism in AD. Sajan et al. (2007) compared the expression of 14 apoptotic genes between normal and AD human hippocampi and noted upregulation in gene expression for c-Fos and BAK in AD patients suggesting the role of these genes in apoptotic cascades of AD. Further Lee et al. (2012) highlighted caspase-independent mode of neuronal death in AD (AIF-induced apoptosis) by studying the expression of AIF protein in different regions of the brain with increased disease progression and noted increased AIF immunoreactivity within the nucleus of hippocampal pyramidal CA1 neurons in early-stage and CA2 in the late stage of AD compared to age-matched, healthy control brains. Colurso et al. (2003) observed a significant degree of DNA fragmentation, dense plaque accumulation and moderate correlation between the two in brain tissues from AD cases compared to controls thus highlighting beta-amyloid as one of the triggers to provoke cell injury in AD. In contrast, no apoptotic morphology was noted in any areas of brain in AD patients, while many ISEL positive nuclei and glia-like cells and necrotic morphology were noted (Lucassen et al., 1997). This set of contrasting observations raises doubts about apoptosis as the main mechanism responsible for neuronal cell death in AD. Table 1 depicts cell death pathways responsible for neuronal death in postmortem AD brains with evidence from various studies.

**TABLE 1:** Cell death pathways responsible for neuronal death in postmortem AD brains with evidence from various studies.

Post-mortem studies in AD brain		
1. Apoptosis	DNA fragmentation and autophagic vacuoles in large number of neurons. Heightened apoptosis with AD. Higher TUNEL staining seen in neurons. Higher caspase 3 levels in neurons from entorhinal cortex, frontal cortex, and hippocampal area. Role of caspase 1, 6, 7, 8, 9 observed. Intracellular Aβ accumulation, p53-mediated transcriptional upregulation of BAX, reduction in bcl-2 and bcl-xl levels. Upregulation of Bad activity in frontal cortical and hippocampal tissue and Casp-3 in cortical neurons.	Smale et al., 1995 Troncoso et al., 1996; Selznick et al., 1999; Stadelmann et al., 1999; Su et al., 2001 Troncoso et al., 1996 Selznick et al., 1999; Stadelmann et al., 1999; Su et al., 2001 Rohn et al., 2001; Matsui et al., 2006; Albrecht et al., 2009; Burguillos et al., 2011 Kitamura et al., 1998 Zhang et al., 2021
2. Non-apoptosis	Cells with swollen morphology and DNA fragmentation imply non-apoptotic mechanism.	Lassmann et al., 1995; Lucassen et al., 1997
A. Autophagy	Accumulation of immature autophagic vacuoles in dystrophic neuritis. Autophagosomes with double membrane encapsulation around intracellular components. Coincidence of downregulated BECN1-PIK 3C3, ULK1/2-ATG13-FIP200, and NRBF2 with AD progression. Transcriptional upregulation of autophagy in entorhinal cortical region.	Nixon et al., 2005 Nixon, 2013 Lachance et al., 2019 Lipinski et al., 2010
B. Necroptosis	Necroptosis activation and its positive correlation with Braak staging; inverse correlation with cognitive scores and brain weight. Co-localization of granular, incipient pTau aggregates with increased chaperone immunoreactivity. Inverse correlation of granulovacuolar degeneration with neuronal density in CA1 region of hippocampus and frontal cortex layer III. Upregulation in MLKL or p-MLKL along with elevated TNF-α? signaling in AD. Link between TNF signaling with necroptosis activation and neuronal loss in CA1-2 hippocampal neurons.	Caccamo et al., 2017 Yamoah et al., 2020 Koper et al., 2020 Xu et al., 2021 Jayaraman et al., 2021
C. Pyroptosis	Enhanced expression of active caspase-1 expression in human MCI and AD brains.	Heneka et al., 2013
D. Parthanatos	Increased Parylation of nuclear proteins. Co-localization of PARP1/PAR with Aβ, Tau and microtubule-associated protein 2. CA1 region showed significant differences in nucleolar PARP-1 staining with Control > AD > MCI cases.	Love et al., 1999 Narne et al., 2017 Regier et al., 2019
E. mPTP	Elevation in CypD expression. Elevated CypD expression in mitochondria.	Du et al., 2008
F. Ferroptosis	Higher cortical iron content in MCI with plaque load, positive correlation of brain iron levels with AD progression and diminution in cognitive ability. Lipid peroxidation and depleted glutathione levels.	Hare et al., 2013; van Bergen et al., 2016; Ayton et al., 2017 Ansari and Scheff, 2010; Yoo et al., 2010; Chiang et al., 2017; Jenkins et al., 2020
**Other human samples**		
Pyroptosis	Upregulated levels of mRNA for inflammasome components (NLRP1, NLRP3, PYCARD, caspase 1, 5 and 8) and downstream effectors (IL-1β, IL-18) in severe and mild AD cases. Increased mRNA and protein levels of caspase-1, NLRP3, GSDMD, and IL-1β in PBMCs of aMCI/AD patients, higher plasma and CSF IL-1β levels in aMCI and AD patients.	Saresella et al., 2016 Rui et al., 2021

##### Necroptosis

Tanaka et al. (2020) in their recent study on MCI vs. AD patients observed an increased necrosis rate in brain tissues from MCI cases as compared to symptomatic AD cases, thus implying that YAP (Yes-associated protein) deprivation-induced neuronal necrosis in early AD stages much before aggregation of extracellular Amyloid beta. In their study on post-mortem human AD brains, Caccamo et al. (2017) reported necroptosis activation and its positive correlation with Braak staging while an inverse correlation was noted with cognitive scores and brain weight. Similar results of necroptosis detection in granulovacuolar degeneration, its association with neuronal loss and correlation with tauopathy and abnormal aggregation of ER chaperones and RNA binding proteins were seen in AD by Yamoah et al. (2020). Koper et al. (2020) in their recent study conducted on brains of preclinical AD patients observed inverse correlation of GVDn+ (granulovacuolar degeneration) with neuronal density in CA1 region of the hippocampus which is early affected and also with late affected frontal cortex layer III and concluded that necrosome represents necroptosis-related neuron death in AD. Consistent with these observations, Xu et al. (2021) also demonstrated upregulated levels of MLKL or p-MLKL in brains of AD patients and concluded that in AD; elevated TNF-α signaling induces necroptosis activation in neurons. Similar findings of link between TNF signaling with activation of necroptosis and loss of neurons in CA1-2 hippocampal neurons have been reported by Anusha Jayaraman et al. (2021) in their recent study on post-mortem brain tissue from AD patients. Based on their observations, the authors hypothesized that in AD; neuronal cell death by TNF-α mediated necroptosis is an early event in disease progression and this signaling pathway is amenable to various therapeutic interventions. In a very recent study, Salvadores et al. (2022) demonstrated association of necroptosis effector pMLKL with Aβ pathology and correlation of Aβo with various markers of necroptosis activation.

##### Autophagy

Nixon et al. (2005) observed accumulation of immature autophagic vacuoles (AV) in dystrophic neurites from AD brain samples by using an electron microscope. A striking increase in classic autophagosomes with double membrane encapsulation around heterogeneous intracellular components was reported in the brains of patients with early stages of AD by the same group several years later (Nixon, 2013). Véronik Lachance, however, reported downregulation of various autophagy kinase complex components, e.g., BECN1-PIK 3C3, ULK1/2-ATG13-FIP200, and NRBF2 and this coincided with clinical dementia progression (CDR) in AD patients (Lachance et al., 2019). Genome-wide analysis of AD patients indicated transcriptional upregulation of autophagy in entorhinal cortex region of brains of AD patients (Lipinski et al., 2010).

##### Parthanatosis

There is increasing evidence of the involvement of PARP-1 in causing a neuronal loss in various neurological diseases, including AD (Fatokun et al., 2014; Narne et al., 2017). Nuclear proteins Parylation was demonstrated in AD human brains (Love et al., 1999). Co-localization of PARP1/PAR with both Aβ, Tau and also microtubule-associated protein 2, which is a dendritic marker was observed in the brain of AD patients (Narne et al., 2017). Regier et al. (2019) examined the expression of PARP-1 in the hippocampi of MCI individuals and compared the same with both control and AD cases and noted decreased PARP-1 immunohistochemical staining in nucleolar region of hippocampal pyramidal cells (CA1 region) in AD and MCI patients than controls and hypothesized that disruption of both form and function of nucleolus in progression of cognitive impairment in MCI and AD cases. Further, the authors suggested decreased immunohistochemical staining of nucleolar PARP-1 in hippocampal pyramidal cells could be considered an early marker of impairment in cognition in AD patients (Regier et al., 2019).

##### Mitochondrial permeability transition pore

Cells can die as a result of mPT and this is another distinctive form of cell death. In an Aβ-rich environment seen in AD, the role of Aβ in formation of mPTP is speculated. CypD is a crucial regulator of mPTP and increased CypD expression favors mPTP activation (Jia and Du, 2021). Hippocampal and Cortical regions of brains of AD patients demonstrated elevated expression of CypD in mitochondria than in those from non-AD brains (Du et al., 2008). A positive association between increased expression of CypD and Aβ was noted by Du and Yan (2010).

##### Ferroptosis

With aging, the cerebral iron levels increase (Bartzokis et al., 2007; Acosta-Cabronero et al., 2016) and this is related to impairment in cognition (Daugherty et al., 2015; Ghadery et al., 2015). Supporting this hypothesis; Tao et al. (2014) in a meta-analysis reported significantly elevated Iron levels in multiple cortical regions of brain tissue from 300 AD cases across 19 studies. Further higher content of cortical iron was observed in MCI patients with plaque load and these brain iron levels positively correlated with progression of AD and diminution of cognition (Hare et al., 2013; van Bergen et al., 2016; Ayton et al., 2017). Iron not only increases toxic Aβ plaque formation (Becerril-Ortega et al., 2014; Ayton et al., 2017; Everett et al., 2018), hyperphosphorylation of tau (Sayre et al., 2000; Hare et al., 2013) but also induces oxidative damage in neurons directly (Thirupathi and Chang, 2019). Lipid peroxidation and depletion in levels of glutathione were noted in post-mortem studies on AD brains (Ansari and Scheff, 2010; Yoo et al., 2010; Chiang et al., 2017; Jenkins et al., 2020). Ashraf et al. (2020), however, demonstrated Iron dyshomeostasis, elevated ferritin levels and the absence of elevated elemental iron in AD patients.

#### Experimental evidence

##### Apoptosis

Results from a recent study by Zhang et al. (2021) show upregulation in the pro-apoptotic activity of Bad in frontal cortical and hippocampal tissues and caspase-3 in cortical neurons in AD brains from mice AD models. The authors also observed restoration of behavioral alteration and cognitive decline in 5X-FAD mice after loss of Bad. This loss of Bad inhibited both neuronal apoptosis and neuronal degeneration and based on their observations, the authors highlighted the role of Bad as a genetic link between apoptosis in neurons and pathology of AD (Zhang et al., 2021). Selznick et al. (1999) in their study on APPsw transgenic mouse noted no association between amyloid-beta deposition with caspase-3 activation contradictory to findings *in vitro*. Qian et al. (2015) studied the effect of caspase-8 knockdown on apoptosis induced by β amyloid in PC12 cells and noted significant inhibition in proliferation of PC12 cells, while caspase 8 siRNA transfection led to a reduction in apoptosis after treatment with β-amyloid. Table 2 depicts cell death pathways responsible for neuronal death in experimental studies on AD models postmortem AD brains with evidence from various studies.

**TABLE 2:** Cell death pathways responsible for neuronal death in experimental studies on AD models with evidence from various studies.

Experimental studies in AD models		
1. Apoptosis	Improvement of behavioral alteration and cognitive decline in 5X-FAD mice after Bad loss.	Zhang et al., 2021
2. Autophagy	Autophagosomes accumulation in neuronal dendrites in PS-1/APP double transgenic mice before amyloid plaque formation. Accumulation of immature AVs in hippocampal neurons of AD mice before actual synaptic and neuronal loss.	Yu et al., 2005 Sanchez-Varo et al., 2012
3. Necroptosis	Reduction in cell loss after lowering necroptosis activation. Co-localization of granular, incipient pTau aggregates in pR5 tau transgenic mouse neurons with increased chaperone immunoreactivity. Upregulated levels of MLKL or p-MLKL (necroptosis marker).	Caccamo et al., 2017 Yamoah et al., 2020 Xu et al., 2021
4. Pyroptosis	Amyloid-β induced upregulation of NLRP1 inflammasome, NLRP1-mediated caspase-1-dependent ‘pyroptosis.’ Protection of Nlrp3^–/–^ or Casp1^–/–^ mice from spatial memory loss and other AD associated sequelae. Aβ_1–42_ induced pyroptosis, increased cell permeability and LDH release, Upregulated cellular GSDMD and p30-GSDMD expression, NLRP3 inflammasome and GSDMD-cleaved protein caspase-1 inflammatory factors levels. Inflammasome-mediated pyroptosis induced by hyperphosphorylated tau.	Tan et al., 2014 Heneka et al., 2013 Han et al., 2020 Li Y. et al., 2020
5. Parthanatos	PARP-1 activation at early stages of amyloid deposit. Crossing of PARP-1 [−/−] mouse with AD transgenic mouse prevented synaptic damage, cognitive dysfunction and microglial activation. p53, PARP-1, NF-κB, Bax overexpression, decreased Bcl-2 levels in Aβ _[1–42]_ treated group while upregulation in Bcl-2 and downregulated PARP-1, NF-κB, p53, and Bax levels with NA treatment. Aβ42 induced hippocampal neurotoxicity by oxidative stress-mediated PARP1 activation, TRPM2 activation, Ca^2+^ influx and mitochondrial dysfunction.	Martire et al., 2013 Kauppinen et al., 2011 Turunc Bayrakdar et al., 2014 Li and Jiang, 2018
6. mPTP	Co-localization of extracellularly applied Aβ with mitochondrial markers in inner mitochondrial membrane. Increased Cyp D levels in AD mouse model with overexpression of mutant human form of APP. Inhibition of ABAD-Aβ interaction, *in vitro* and *in vivo*, protected against aberrant mitochondrial and neuronal function, improved spatial learning/memory. Increased CypD expression in cortical mitochondria with translocation to IMM, Ca^2+^ dyshomeostasis and mitochondrial swelling. Mitochondria derived from CypD-deficient mAPP mouse model (mAPP/Ppif^–/–^) exhibited reduction in mitochondrial swelling as well as permeability transition after induction by Ca^2+^. Aβ accumulation provoked Ca^2+^ overload in mitochondria via MCU complex. Loss of efflux from MCU led to mCa^2+^ overload, promoted superoxide generation, metabolic dysfunction and neuronal cell death.	Hansson Petersen et al., 2008 Du et al., 2008 Yao et al., 2011 Hung et al., 2010 Du et al., 2008; Yao et al., 2011 Calvo-Rodriguez et al., 2020 Jadiya et al., 2019
7. Ferroptosis	Spatial learning and memory function deficits co-related with lipid peroxidation, ERK activation and increased neuroinflammation.	Hambright et al., 2017

##### Necroptosis

Caccamo et al. (2017) in their study on a mouse model of AD, observed a reduction in cell loss when necroptosis activation was lowered and hypothesized that new therapeutic strategies blocking necroptosis activation hold strong potential in prevention and treatment of AD. Another group demonstrated that necrostatin-1 treatment in APP/PS1 double transgenic mice led to reduced levels of Aβ-oligomer, hyperphosphorylated tau and amyloid plaque, alleviated brain cell death and also ameliorated cognitive impairments (Yang et al., 2017). Upregulated levels of necroptosis markers (MLKL and p-MLKL) were noted in AD mouse models of APP/PS1 and 5xFAD mice by Xu et al. (2021) and they hypothesized that elevated TNF-α signaling in AD induce activation of neuronal necroptosis.

##### Autophagy

Several studies conducted on various AD models reveal that compromised mitophagy contributes to AD pathology by increasing oxidative stress, synaptic dysfunction, loss of neurons and decline in cognition. Accumulation of autophagosomes in neuronal dendrites was noted by Yu et al. (2005) in PS-1/APP double transgenic mice, however, this abnormal gathering of autophagosomes was found to occur before the formation of amyloid plaque. Similarly, another study reported accumulation of immature AVs in hippocampal neurons of AD mice much before the actual neuronal and synaptic loss (Sanchez-Varo et al., 2012). Correia et al. (2015) stated that deficiency(ies) in autophagosome pathway precedes the formation of Aβ plaques or neurofibrillary tangles in AD. Lachance et al. (2019) demonstrated that deletion of NRBF2 (a component of the BECN1-PIK3C3 complex) reduced autophagy, led to impairment of memory in mice, altered long-term potentiation (LTP) and promoted accumulation of Aβ. Furthermore, overexpression of NRBF2 in the hippocampus rescued impaired autophagy and memory deficits and also reduced β-amyloid levels and improved memory in an AD mouse model (Lachance et al., 2019).

##### Pyroptosis

Mounting evidence suggests activation of NLRP3 inflammasome in AD and its role in causing pathological changes in AD mice through the pyroptosis pathway (Heneka et al., 2013; Abbott, 2018). While upregulation of NLRP3, a key player of pyroptosis was observed in neurons of AD mouse models, its knockdown led to reduced neuronal death and also reversed cognitive impairment (Tan et al., 2014). Aβ and hyperphosphorylated tau (the pathological hallmark of AD) have been shown to activate the inflammasomes (NLRP1, AIM2 and NLRP3) and lead to GSDMD-mediated neuronal pyroptosis both *in vitro* and *in vivo* (Heneka et al., 2013; Tan et al., 2014; Han et al., 2020). Li Y. et al. (2020) evaluated the reciprocity between hyperphosphorylated tau and pyroptosis by using *in vitro* (FSK or STZ-treated PC12 cells) and *in vivo* (ICV-FSK or STZ rat) models and based on their observations, the authors concluded that inflammasome-mediated pyroptosis is one of the underlying mechanism responsible for neurotoxicity induced by hyperphosphorylated tau in both the AD models used. Rui et al. (2021) in a recent study observed increased levels of both mRNA and proteins of caspase-1, NLRP3, GSDMD, and IL-1β in PBMCs of aMCI/AD patients as compared to controls. Plasma and CSF IL-1β levels were found to be significantly higher in aMCI and AD patients and it correlated negatively with Aβ_1–42_ levels in CSF and with MMSE and MoCA scores. Based on these findings the authors inferred that there is peripheral activation of NLRP3/caspase-1/GSDMD signaling pathway in aMCI and AD patients and concluded that targeting inflammasome induced pyroptosis might serve as a novel approach to mitigate neuroinflammation seen in these patients (Rui et al., 2021). Shen et al. (2021) noted higher levels of GSDMD, t-tau and tau 181p in CSF samples of AD patients as compared to controls and highlighted good diagnostic accuracy of GSDMD in AD. Further, Saresella et al. (2016) in their study on PBMC from MCI, moderate and severe AD and healthy controls observed upregulated mRNA levels for various components of inflammasome and IL-1β, IL-18 in patients with severe and mild AD. To further support this set of observations; Heneka et al. (2013) in their study on APP/PS1 mice demonstrated that NLRP3^–/–^ and Casp1^–/–^ mice were protected from spatial memory loss and other AD sequelae.

##### Parthanatosis

Li and Jiang (2018) in their study on hippocampal mice neurons; demonstrated that amyloid β led to hippocampal neurotoxicity by inducing oxidative stress-mediated PARP1 activation, transient receptor potential melastatin-related 2 (TRPM2) activation and further causing Ca^2+^ influx and mitochondrial dysfunction. Martire et al. (2013) in their series of experiments on neuroblastoma cells noted that treatment with Aβ_25–35_ led to enhanced PARP activity which was seen to be prevented by pre-treatment of cells with MC2050 (PARP-1 inhibitor). Similar results were observed in CHO cells and brain specimens from TgCRND8 transgenic mice, thus confirming the activation of PARP-1 by Aβ_25–35_ induced DNA damage. Further Aβ25–35 activated NF-kB, upregulated p53 protein levels along with a decrease in Bcl-2 protein (Martire et al., 2013). Turunc Bayrakdar et al. (2014) in their study on four groups of Sprague–Dawley rats control, Aβ_(1–42_), Aβ_(1–42)_ + NA (nicotinamide 100 and 500 mg/kg), noted overexpression of p53, PARP-1, NF-κB, Bax levels and decreased Bcl-2 levels in Aβ_(1–42)_-treated group while upregulation in Bcl-2 and downregulated PARP-1, NF-κB, p53, and Bax levels was noted with NA treatment. Crossing of PARP-1 (−/−) mouse with AD transgenic mouse could prevent the development of synaptic damage, cognitive dysfunction and also activation of microglia. Furthermore, it was observed that when PARP-1 (−/−) mice were injected with Aβ or when PARP-1 was inhibited with PJ34; lower microglial activation was induced as compared to controls. Based on these observations, the authors concluded that PARP-1 inhibition or KO improved synaptic damage, cognitive dysfunction and microglial activation (Kauppinen et al., 2011).

##### Mitochondrial permeability transition pore

The formation of mPTP is a mitochondrial response to both intramitochondrial as well as intracellular stresses (Jia and Du, 2021). In a study by Hansson Petersen et al. (2008) on isolated rat mitochondria; co-localization of extracellularly applied Aβ was observed with mitochondrial markers in IMM, thus suggesting that Aβ was taken by the cells. An increase in Cyp D levels was noted in an AD mouse model with overexpression of mutant human form of APP; highlighting the fact that Aβ acts as a mediator connecting AD to mPTP (Du et al., 2008). Cyp D deficiency in transgenic mice overexpressing APP and Aβ improved both synaptic and mitochondrial function and learning/memory (Du et al., 2011). The interplay between intramitochondrial Aβ and CypD results in the formation of mPTP; which reduces mitochondrial potential, decreases mitochondrial respiratory function, and increases formation of free radicals that causes oxidative stress and releases pro-apoptotic proteins (Du et al., 2008; 2011; Yao et al., 2011; Rao et al., 2014). Research using mAPP model of mice has established that mPTP formation is accompanied by increased CypD expression in cortical mitochondria with translocation to IMM, Ca^2+^ dyshomeostasis and mitochondrial swelling (Du and ShiDu Yan, 2010; Hung et al., 2010; Rao et al., 2014). Further to support this observation, it was noted that CypD ablation with cyclosporine A (CsA), mitigated these detrimental effects (Du et al., 2008; Yao et al., 2011). Similarly, significant improvement in learning/memory and mitochondrial function was observed by Du et al. (2008, 2011) in their study on CypD deficient mice thus highlighting the fact that Cyp-D ablation in mice protects them from behavioral and mitochondrial dysfunction throughout their lives. Mitochondria derived from CypD-deficient mAPP mouse model (mAPP/Ppif−/−) exhibited a reduction in mitochondrial swelling as well as permeability transition after induction by Ca^2+^ compared with mAPP mice. In a recent study on a transgenic mouse model of cerebral β-amyloidosis, it was demonstrated that accumulation of Aβ oligomers provokes Ca^2+^ overload in mitochondria via the MCU Complex (mitochondrial Ca^2+^ uniporter), thus contributing to neuronal death (Calvo-Rodriguez et al., 2020). In another study; Jadiya et al. (2019) revealed that loss of efflux from MCU leads to mCa^2+^ overload which accelerates the development of AD by promoting generation of superoxide, metabolic dysfunction and neuronal cell death.

##### Ferroptosis

In a series of experiments by Hambright et al. (2017) on GPX4BIKO mice, defects in both spatial learning and memory function were observed and were related to lipid peroxidation and increased neuroinflammation while liproxstatin-1 treatment improved neurodegeneration in these mice. Bao et al. (2021) reported downregulation of Ferropotin1 (Fpn-mammalian non-heme iron exporter) in brains of APPswe/PS1dE9 mice and genetic deletion of Fpn led to AD-like atrophy in the hippocampus region along with various memory deficits.

**FIGURE 1:**
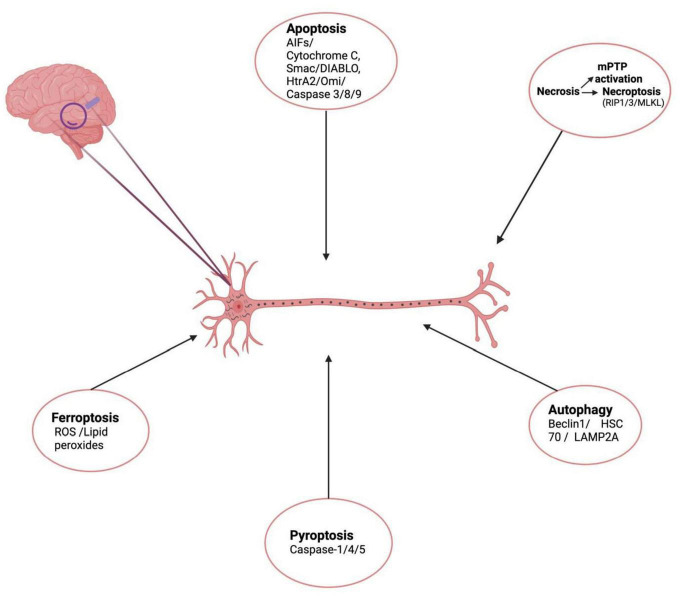
Schematic representation of cell death mechanisms responsible for neuronal death in AD. (Created with BioRender.com.) Apoptosis – initiated inner mitochondrial membrane changes leads to release of cytochrome c, AIF, Smac/DIABLO, HtrA2/Omi, activates effector caspases and causes apoptotic cell death. Necroptosis – is a form of RCD involving RIPK1, RIPK3, necrosome formation and MLKL activation. Another form of necrosis mPTP leading to rupture of outer membrane and non-specific release of intermembrane space proteins into cytosol, causing cell death. Autophagy – another cell death mechanism mediated by Beclin 1, HSC70, LAMP2A leads to formation of LC3II autolysosomes causing cell death. Pyroptosis – lytic form of cell death associated with inflammasomes formation, activation caspase-1/4/5 Gasdermin-D cleavage and release of inflammatory cytokines is also responsible for neuronal cell death in AD. Ferroptosis – Iron-dependent mode of cell death, induced by diverse triggers involves ROS, lipid peroxide accumulation depleted glutathione levels.

Considering the complex cellular and architectural diversity of brain, it is very reasonable to foresee that not one but multiple forms of cell death occur simultaneously and are responsible for AD pathogenesis (Moujalled et al., 2021). Complex interplay among these RCD cascades and it is this crosstalk between these processes that stand out as the main cause of neuronal death in AD and other neurodegenerative diseases (Saleem, 2021). Adrienne M. Gorman in a review stated that more than one type of cell death mechanism can be induced by multiple intracellular and extracellular cell death stimuli depending on several conditions such as duration and severity of stress, the health status (redox levels, integrity of mitochondria) within the cell milieu, etc. (Gorman, 2008). To maintain cellular homeostasis in response to several stressors, the neurons respond by enhancing cross-talk between various cell death pathways. Initially as a pro-survival mechanism to stressors; the cells perform autophagy and avoid cell death but when the level of stressors increases beyond a limit, autophagy is blocked while apoptotic cascades are initiated (Wu et al., 2014). Cell death becomes the only solution when the stress signals cross a certain threshold level where recovery becomes impossible. Autophagy has been reported to act as a switch that regulates cell death by shifting between necrosis and apoptosis (Goodall et al., 2016). In recent studies many key players like Beclin1, Atg1, p53, RIPK1, LC3, p62, TRIB3 play a pivotal role in switching between these death paradigms by toggling from one to the other cell death mechanism, cross communicating between various cell death pathways thus creating a complex scenario of cell death in various neurodegenerative diseases including AD (Saleem, 2021). Beclin-1 which is autophagy associated protein has been shown to interact with Bcl-2, undergo modifications and induce apoptosis and inhibit autophagy (Wang et al., 2018). Studies demonstrate induction of autophagy by pro-apoptotic protein, p53 upon proteasomal inhibition (Du et al., 2009; Dong et al., 2012). The role of p62 which is an autophagy flux marker has been suggested in apoptosis. Another protein Trib-3 (pseudo-kinase) has been shown to induce both autophagy and apoptosis. Similarly, TNF-α might cause simultaneous activation of both RIPK1-dependent apoptosis and necroptosis with interactions between each pathway (Geng et al., 2017). Considering the complex cellular and architectural diversity of the brain, it is very reasonable to foresee that not one but multiple forms of cell death occur simultaneously and are responsible for AD pathogenesis (Moujalled et al., 2021). Figure 1 depicts the schematic representation of cell death mechanisms responsible for neuronal death in AD.

Results from a study on age-associated changes in OXYS rats at different ages reveal that the nature of alterations in various RCD pathways as outlined above depends on the stage of AD. Based on their observations, the authors concluded that during late stage of AD-like pathology; apoptosis and necroptosis gets activated because of a decline in autophagy-mediated proteostasis (Telegina et al., 2019). Since the development of AD starts significantly before the patient/s present clinically; Morsch et al. (1999) proposed that though RCD activation and neuron loss continue to take place in AD, still neurons with AD pathology continue to survive for decades. Subsequently, it is therefore projected that besides RCD activation, there is an activation of compensatory pathways within the neurons to counteract the various neurotoxic effects of the disease (Bloss et al., 2008; Okonkwo et al., 2012; Talboom et al., 2019). Multiple such cell survival pathways which promotes cell repair, inhibit cell death and respond to cytotoxic stress might be involved in compensation. These pathways include the PI3K/AKT that promotes cell survival and inhibits apoptosis (Heras-Sandoval et al., 2014; Morita et al., 2015), the MAPK pathway (Kim and Choi, 2015), and NF- κB pathway that promote cell survival by various direct and indirect mechanisms (Nikoletopoulou et al., 2013; Kim and Choi, 2015; Pires et al., 2018). These signaling pathways might on one hand promote cell survival and mitigate cell stress in AD but on the other hand; their hyperactivation might lead to neuropathology as seen in AD (Heras-Sandoval et al., 2014; Kim and Choi, 2015; Shi et al., 2016).

## Triggers for neuronal death in Alzheimer’s disease

### Protein aggregates

Cell death emerges as the final solution for a neuron when multiple stresses pile up to such an extent that is much beyond the healing capacity of the cell and this point acts as a trigger for activation of RCD signaling cascades ultimately resulting in the development of a multitude of neurodegenerative diseases including AD. Most of neurodegenerative diseases are linked with formation/accumulation of pathological protein(s) to the extent of formation of high-order aggregates (Soto, 2003; Blokhuis et al., 2013; Michel et al., 2016). These higher-order aggregates act as stressors and induce a plethora of cytotoxic mechanisms including upregulation in ROS levels, synaptic dysfunction, excitotoxicity, impaired degradation of protein systems, ER stress, inflammation, cell cycle re-entry, DNA damage and mitochondrial dysfunction ultimately culminating in neuronal cell death (Seward et al., 2013; Currais et al., 2016; Chi et al., 2018).

#### Amyloid beta and its role in neuronal cell death

Hardy and Selkoe in the year 2002 proposed the “amyloid cascade hypothesis” as the main theoretical construct for AD in which Aβ peptide was postulated as the direct causative agent responsible for progressive neurodegeneration. (De Strooper and Karran, 2016) Recently the “Aβ oligomer hypothesis” has supplanted this “amyloid cascade hypothesis” and provides a wealth of insights on the fact that it is AβOs and not amyloid plaques that play a central role in AD pathogenesis (Selkoe and Hardy, 2016). The collective evidence from various behavioral, neuropathological, and cell biological studies available in literature reveal that elevated levels of AβOs in the brain have pathogenic consequences (Cline et al., 2018). The oligomeric and fibrillar species of Aβ formed after its aggregation is toxic. After the initial transduction; downstream the AβOs have multiple effects on different subcellular organelles and have multiple effects including mitochondrial effects, ER stress, and autophagy/lysosomal dysfunction (Cline et al., 2018). Impairment in Aβ processing pathways leads to its deposition which adversely affects a large number of pathways such as neurotransmitter release, intracellular signaling cascades, autophagy regulation, lipid metabolism, and synaptic function culminating in neuronal death (Serpell et al., 2000). Soluble Aβ oligomers and insoluble aggregates of Aβ bind and activate microglia and astrocytes and stimulate a low level of chronic neuroinflammation. Furthermore, APP accumulation leads to an increase in intracellular calcium, which causes excitotoxicity, as well as an increase in pro-apoptotic molecules and heat shock proteins (Mattson et al., 1992). Multiple mechanisms induced by intraneuronal Aβ oligomers have been postulated for the etiopathogenesis of AD including ER stress-induced apoptosis (Nishitsuji et al., 2009), endosomal/lysosomal leakage, mitochondrial dysfunction (Umeda et al., 2011), oxidative stress (Tabner et al., 2005; De Felice et al., 2007), deterioration of synapses (Lacor et al., 2007; Shankar et al., 2007). Further Zhao et al. (2008, 2009) demonstrated that Aβ oligomers induce insulin resistance in AD brains while brain insulin and IGF-1 receptors dysfunction further lead to Aβ aggregation and synaptic loss. Ferretti et al. (2012) suggested neuroinflammation as one of the earliest pathological responses to intracellular Aβ-oligomers accumulation. Tomiyama et al. (2010) in their experiment on APP transgenic mice with E693Δ mutation observed that intraneuronal Aβ oligomers accumulation not only led to impairment of hippocampal synaptic plasticity and memory, downregulation of presynaptic protein synaptophysin but also abnormal phosphorylation of tau, activation of microglia and astrocyte with neuronal loss, thus highlighting that Aβ oligomers activate multiple causal mechanisms responsible for the pathogenesis of AD.

#### Tau

The second core pathology in AD is the formation of neurofibrillary tangles (NFT) that are formed from tau hyperphosphorylation. Tau protein gets phosphorylated at multiple sites in AD and tau gets removed from the microtubule resulting in collapse of microtubule structure and disruption of various cellular processes. Further p-tau aggregates and forms paired helical fragments and eventually, NFTs are formed. This pathogenic form of Tau at the molecular level acts as a stressor for neurons in multiple ways (Chang et al., 2018). But how tau can directly induce neuronal death is yet unanswered. Hyperphosphorylated tau accumulation alters microtubule stability, resulting in two outcomes: (1) tau interacts with synaptogyrin-3 (a pre-synaptic protein) leading to defects in synaptic release, synaptic loss and neuronal dysfunction (McInnes et al., 2018) and (2) activation of retrograde neurodegeneration and neuronal death. Tau aggregation has also been found to be linked to stocktickerDNA fragmentation, implying a relationship to regulated neuronal cell death. Further at the post-synaptic level; Tau stabilizes NMDA receptors and has been shown to elicit excitotoxicity (Ittner et al., 2010, 2016). Another study discovered that phosphorylation of numerous particular Tau residues causes cyclin D1 and BrdU incorporation to increase (Zhao et al., 2003). Prolonged NMDA receptor activation incites Ca^2+^, activates calpain and causes mitochondrial dysfunction leading to cell death (Bano and Ankarcrona, 2018). Furthermore, evidence from several studies indicate that tau pathology affected several systems within the cell including signaling system (Cabrales Fontela et al., 2017; Zhou et al., 2017; Bardai et al., 2018), transport system, cytoskeletal system (Fulga et al., 2007) and also disturbed mitochondrial integrity (DuBoff et al., 2012) resulting in extensive damage within the neurons.

Furthermore in a recent *in vivo* study; Pereira et al. (2021) demonstrated toxic effects of amyloid-β and tau on synaptic function and axonal integrity respectively in the etiopathogenesis of AD. The authors showed that early stages of AD are characterized by synaptic damage induced by amyloid deposits, impaired memory and changes in functional connectivity while tau-associated damage to axons, cognitive decline and anatomical connectivity reduction is seen in late stages of AD.

### Inflammation and microglial response

Inflammation in Alzheimer’s disease plays a role in both the onset as well as progression of the disease. The presence of amyloid β has been proposed to be the primary driver for microglia activation as intracellular Aβ accumulation triggers a neuron-derived inflammatory reaction, characterized by mobilization of activated microglia toward damaged site (Welikovitch et al., 2020). A buildup of glial cells that normally keep brain debris-free results in chronic inflammation. In AD, microglia fail to remove debris and various protein aggregates including amyloid β, become chronically activated and release various pro-inflammatory and toxic products including cytokines, ROS, NO, etc. The expression of various inflammatory cytokines is higher in the vicinity of NFT and Aβ peptide deposits (Akiyama et al., 2000). Multiple studies reveal inflammatory response and activation of microglia in both post mortem tissues obtained from AD patients (Fan et al., 2015; Stefaniak and O’Brien, 2016; Passamonti et al., 2019), and in various preclinical AD models (Heppner et al., 2015; Hoeijmakers et al., 2016; Villegas-Llerena et al., 2016). Hong et al. (2016) in their study on AD mouse models demonstrated that the challenge of synaptotoxic Aβ oligomers results in inappropriate activation of microglia in adult CNS which phagocytose the synapses and causes synaptic loss and concluded that immune-related pathways and microglia act as early mediators for loss of synapse and its dysfunction much before plaque formation. Further several Genome-wide association studies state role of microglia-related molecular pathways in the etiopathogenesis of AD. TREM2 mutations on microglia are linked with an increased AD risk (Guerreiro et al., 2013; Jonsson et al., 2013). Passamonti et al. (2019) in a recent study provided clear evidence of the association of impairment in large-scale network connectivity with *in vivo* neuro-inflammation in AD and the degree of this association correlated with a cognitive deficit in the subjects enrolled in the study. Cognitive scores of patients correlated to brain connectivity pattern which was related to microglia activation (Passamonti et al., 2019). Rangaraju et al. (2018) compared the relevance of homeostatic and Disease-associated microglia (DAM) and noted positive association of pro-inflammatory DAM proteins with neuropathology while there was high representation of human AD risk genes within homeostatic microglia.

## Open questions and conclusion

This review has clearly brought out the knowledge-gap in our understanding of the mechanisms of neuronal death in clinical cases of AD. While clearly defined RCD pathways have been demonstrated in a large number of studies in experimental AD models, it is questionable if these can be extrapolated directly to clinical AD. This is particularly because many of these studies employed transgenic AD animals which could not be considered as representatives of vast majority of AD patients who suffer from the sporadic form of the disease. On the other hand, post-mortem studies present the end-points of the pathology and fail to establish the ante-mortem scenario of neuronal death in the AD brain; this is further complicated by the post-mortem artifacts and various limitations of biochemical and immunohistochemical methods applicable to clinical samples to identify clearly the different RCD pathways. It will not be possible to resolve these issues easily, and there are other unanswered questions. For example, in experimental studies *in vitro* the same trigger depending on the context often leads to different RCD pathways, and there also exists extensive cross-talk among these pathways. Likewise, the inflammation, mitochondrial dysfunction and oxidative stress, the key elements of AD pathology, are linked to multiple RCD pathways. Thus, it cannot be ruled out that different neuronal populations in the AD brain may follow different pathways of death. The development of suitable imaging techniques to identify neuronal death in the ante-mortem brain can provide some answers to these questions. Finally, it is important to point out that the attempts to develop neuroprotective therapy for AD have not really targeted RCD pathways; it has mostly targeted the triggers like amyloid beta, oxidative stress, inflammation, etc., by various means without much success in clinical trials. It remains an open question if specific inhibitors of apoptosis, necroptosis or ferroptosis would provide clinical benefit in AD.

## Author contributions

PG and SC provided the idea and mainly wrote the manuscript. PG and KG contributed to the search and assessment of the available literature. All authors contributed to the article and approved the submitted version.
